# Cost-effectiveness of implementing a genotype-guided de-escalation strategy in patients with acute coronary syndrome

**DOI:** 10.1093/ehjcvp/pvae087

**Published:** 2024-11-13

**Authors:** Wout Willem Antoon van den Broek, Jaouad Azzahhafi, Dean R P P Chan Pin Yin, Niels M R van der Sangen, Shabiga Sivanesan, Lea M Dijksman, Ronald J Walhout, Melvyn Tjon Joe Gin, Nicoline J Breet, Jorina Langerveld, Georgios J Vlachojannis, Rutger J van Bommel, Yolande Appelman, Ron H N van Schaik, José P S Henriques, Wouter J Kikkert, Jurriën M ten Berg

**Affiliations:** Department of Cardiology, St. Antonius Hospital, Nieuwegein, The Netherlands; Department of Cardiology, St. Antonius Hospital, Nieuwegein, The Netherlands; Department of Cardiology, St. Antonius Hospital, Nieuwegein, The Netherlands; Department of Cardiology, Amsterdam UMC, University of Amsterdam, Amsterdam Cardiovascular Sciences, Amsterdam, The Netherlands; Department of Cardiology, Amsterdam UMC, University of Amsterdam, Amsterdam Cardiovascular Sciences, Amsterdam, The Netherlands; Department of Value-Based Healthcare, St. Antonius Hospital, Nieuwegein, The Netherlands; Department of Cardiology, Hospital Gelderse Vallei, Ede, The Netherlands; Department of Cardiology, Rijnstate Hospital, Arnhem, The Netherlands; Department of Cardiology, Gelre Hospitals, Apeldoorn, The Netherlands; Department of Cardiology, Rivierenland Hospital, Tiel, The Netherlands; Department of Cardiology, University Medical Center Utrecht, Utrecht, The Netherlands; Department of Cardiology, Tergooi Hospital, Hilversum, The Netherlands; Department of Cardiology, Amsterdam UMC, VU University, Amsterdam Cardiovascular Sciences, Amsterdam, The Netherlands; Department of Clinical Chemistry, Erasmus MC—University Medical Center, Rotterdam, The Netherlands; Department of Cardiology, Amsterdam UMC, University of Amsterdam, Amsterdam Cardiovascular Sciences, Amsterdam, The Netherlands; Department of Cardiology, Amsterdam UMC, University of Amsterdam, Amsterdam Cardiovascular Sciences, Amsterdam, The Netherlands; Department of Cardiology, Tergooi Hospital, Hilversum, The Netherlands; Department of Cardiology, St. Antonius Hospital, Nieuwegein, The Netherlands; Cardiovascular Research Institute Maastricht, University Medical Center Maastricht, Maastricht, The Netherlands

**Keywords:** ACS, Coronary artery disease, P2Y12-inhibitor, Genotype-guided, Cost-effectiveness

## Abstract

**Aims:**

A genotype-guided P2Y12-inhibitor de-escalation strategy, switching acute coronary syndrome (ACS) patients without a CYP2C19 loss-of-function allele from ticagrelor or prasugrel to clopidogrel, has shown to reduce bleeding risk without affecting the effectivity of therapy by increasing ischaemic risk. We estimated the cost-effectiveness of this personalized approach compared to standard dual antiplatelet therapy (DAPT; aspirin plus ticagrelor/prasugrel) in the Netherlands.

**Methods and results:**

We developed a 1-year decision tree based on results of the FORCE-ACS registry, comparing a cohort of ACS patients who underwent genotyping with a cohort of ACS patients treated with standard DAPT. This was followed by a lifelong Markov model to compare lifetime costs and quality-adjusted life years (QALYs) for a fictional cohort of 1000 patients. The cost-effectiveness analysis was performed from the perspective of the Dutch healthcare system. A genotype-guided de-escalation strategy led to an increase of 57.73 QALYs and saved €808788 compared to standard DAPT based on a lifetime horizon. Probabilistic sensitivity analysis showed that the genotype-guided strategy was cost-saving in 96% and increased QALYs in 87% of simulations. The intervention remained cost-effective in the scenario where prices for all P2Y12 inhibitors were equalized. The genotype-guided strategy remained dominant in various other scenarios and sensitivity analyses.

**Conclusion:**

A genotype-guided de-escalation strategy in patients with ACS was both cost-saving and yielded higher QALYs compared to standard DAPT, highlighting its potential for implementation in clinical practice.

**Trial registration:** ClinicalTrials.gov identifier: NCT03823547.

## Introduction

The default antiplatelet treatment in patients with acute coronary syndrome (ACS) is dual antiplatelet therapy (DAPT), comprising aspirin and a potent P2Y12 inhibitor (ticagrelor or prasugrel) for 12 months.^1^ Its goal is to mitigate ischaemic risk, albeit with an associated increase in bleeding risk.^2^ With advancements in secondary prevention and stent technology, ischaemic risk has decreased, opening the door for new strategies that minimize bleeding risk without compromising the reduction of ischaemic risk.^2,3^ The POPular Genetics trial showed in a randomized setting that a *CYP2C19* genotype-guided de-escalation strategy reduced the risk of bleeding without affecting ischaemic risk, compared to standard DAPT in patients with ST-elevation myocardial infarction.^4^ This de-escalation strategy involves switching from the more potent drugs ticagrelor or prasugrel to the less potent clopidogrel in patients without a *CYP2C19* loss-of-function allele. By implementing this strategy, theoretically, 70% of patients who would otherwise receive ticagrelor can instead be treated with clopidogrel, a drug significantly more affordable than ticagrelor and prasugrel.^5,6^ Accordingly, the cost-effectiveness analysis (CEA) of the POPular Genetics demonstrated that a genotype-guided de-escalation strategy is both cost-saving and increases quality of life (QoL).^7^ While randomized clinical trials (RCTs) are crucial for establishing evidence-based foundations for new interventions, the question remains whether results mirror real-world outcomes, where populations are often at higher risk and adoption rates may be lower. Whether the implementation of routine genetic *CYP2C19* testing of ACS patients to guide the selection of the P2Y12 inhibitor is cost-effective compared to standard DAPT remains uncertain. In this analysis, we aimed to assess the cost-efficacy of a genotype-guided de-escalation strategy directly after hospital admission, compared to standard DAPT based on real-world data.

## Methods

### Study design

For this analysis we used data from the FORCE-ACS registry (NCT03823547), of which the rationale and design have been described previously.^8^ In brief, the FORCE-ACS registry is an ongoing, prospective, multicentre registry involving nine Dutch hospitals, consecutively enrolling adult patients with (suspected) ACS since 2015. It's primary objective is to gain insight into the various aspects of care for ACS patients. Before 2021, all local protocols recommended the use of DAPT with a more potent P2Y12-inhibitor (ticagrelor or prasugrel) as the default strategy in ACS patients without an indication for anticoagulation. Since 2021, one hospital (St. Antonius Hospital, Nieuwegein, The Netherlands) has implemented a genotype-guided P2Y12-inhibitor de-escalation strategy in its ACS protocol. At admission, all ACS patients underwent CYP2C19 genotype testing, either through point-of-care testing (POCT) using the Cube CYP2C19 System (Genomadix) or through lab-based testing with the StepOnePlus^TM^ Real-Time PCR system (Applied Biosystems, Thermofisher Scientific). In non-carriers of a *CYP2C19* loss-of-function allele (normal metabolizers), the recommendation was to switch from ticagrelor/prasugrel to clopidogrel. Patients who carried a *CYP2C19* loss-of-function, remained on their current treatment with ticagrelor/prasugrel. Approval was obtained from institutional review boards, adhering to the Declaration of Helsinki and reporting results per STROBE guidelines.

### Population

Patients enrolled in the FORCE-ACS registry were divided into two cohorts: a standard care cohort, in which patients were treated with a P2Y12 inhibitor (ticagrelor, prasugrel, or clopidogrel) at the discretion of the treating physician, and a genotyped cohort, in which patients received a CYP2C19 genotype test with a treatment recommendation based on the result. For the current model, we used the propensity score-matched population from the FORCE-ACS registry, which has been published previously (Supplementary material online, *Table S1*).^9^ This allowed for adjustment of multiple baseline characteristics, yielding two cohorts that were comparable regarding age, medical history, and comorbidities. The median age of the trial population was 64 years old, 28% female and 14% had a prior history of myocardial infarction (MI).

### Model overview

**Figure 1:**
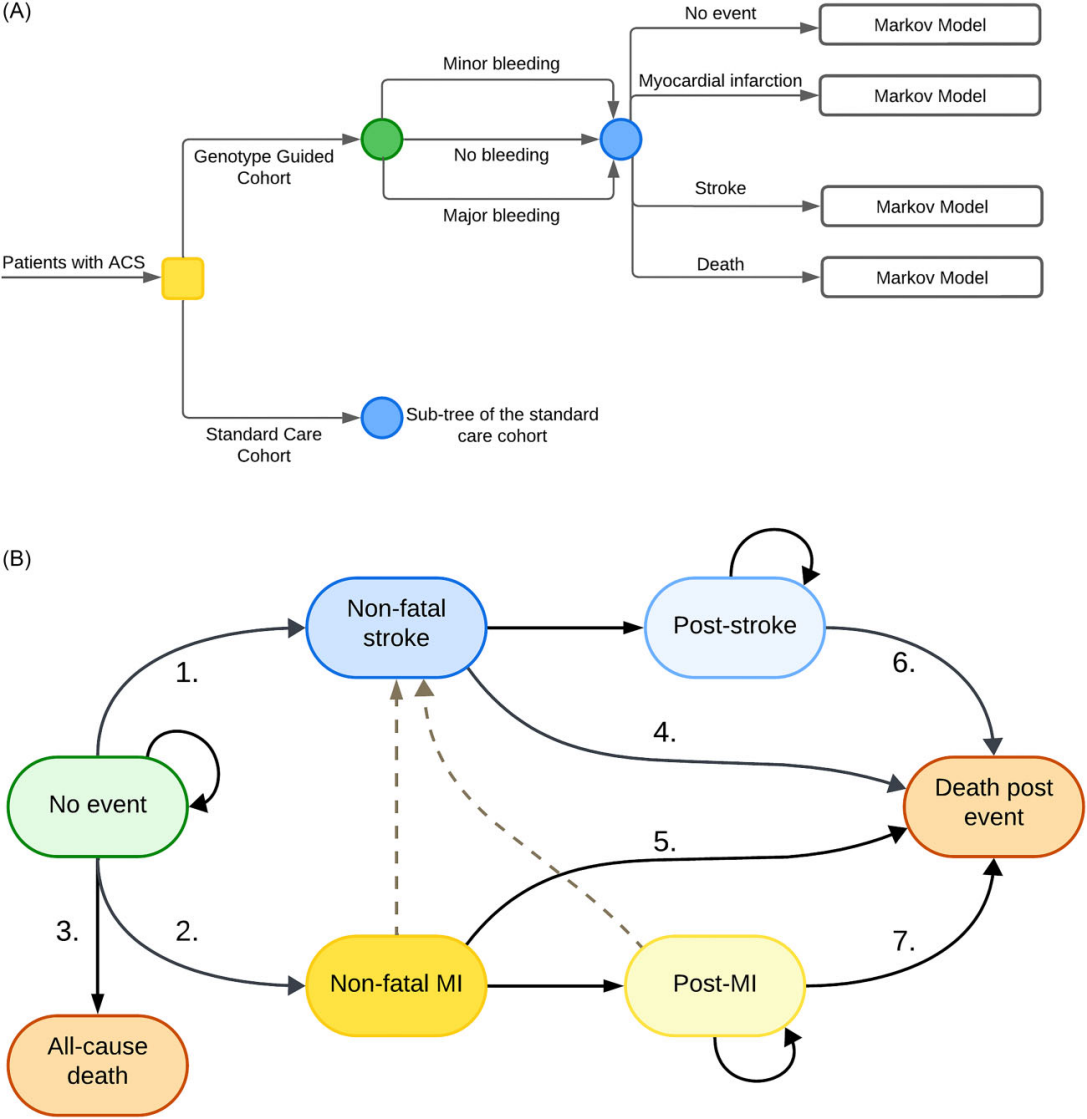
Cost-effectiveness model. (*A*) One-year decision tree. ACS; acute coronary syndrome. (B) Long-term Markov model. Markov model transitions in figure: (1) risk of non-fatal stroke based on literature. (2) Risk of non-fatal MI based on literature. (3) Mortality risk for patients with no event based on Dutch population data. (4) Mortality risk after a non-fatal stroke. (5) Mortality risk after a non-fatal MI. (6) Mortality risk at second and subsequent years after a non-fatal stroke. (7) Mortality risk at second and subsequent years after a non-fatal MI. MI; myocardial infarction. The dotted lines indicate the transition of patients in the non-fatal MI or post-MI state to the non-fatal stroke state.

We developed a two-part decision-analytic model: a 1-year decision tree to allocate patients across Markov states (*Figure 1A*), followed by a Markov model to simulate lifelong costs and effects (*Figure 1B*). All individuals in the hypothetical cohort were at the age of 64 at the start of the model. In the decision tree, all patients had the possibility of experiencing minor or major bleeding, irrespective of other events. Throughout the initial year, patients who experienced a MI or stroke transitioned into corresponding health states, while patients who passed away entered the all-cause death state; all remaining patients entered the no-event state. Following the 1-year decision tree period, patients transitioned between different Markov states based on different transition probabilities. These health states comprised the no event, non-fatal stroke, non-fatal MI, post-stroke, post-MI, and all-cause death states, reflecting the lifetime progression of patients after ACS. The non-fatal MI and non-fatal stroke states were termed ‘tunnel states’, indicating that patients could only remain in each state for one cycle. The structure of the Markov model was aligned with previously published and clinically validated models.^10,11^ A hypothetical cohort of 1000 patients was used to simulate progression and transitions across various health states. In the base case analysis, the lifetime horizon was set at the age of 100 years.

### Model assumptions

We made the assumption that bleeding risk after the 1-year follow-up was comparable in both groups, as patients in both groups were assumed to be treated with aspirin, in line with current ESC guidelines and local protocols.^12^ Because use of oral anticoagulants was rare and comparable between groups, we did not expect this to impact the bleeding rates in the Markov model after the first year. In line with previously published literature, bleeding was not included as a separate health state in the Markov model and decreased QoL for only a short period.^10^ Patients could not develop multiple events during one cycle and could only experience a recurrent stroke or MI with a minimum interval of 1 year.

### Model input parameters

#### Transition probabilities

Probabilities for the distributions in the 1-year decision tree were derived from the propensity score-matched results of the clinical implementation of the genotype-guided strategy.^9^ After constructing the decision tree, patients were assigned to their respective health state in the long-term Markov model. The Markov model, with yearly cycles, simulated disease progression over their lifetime. Patients in each health state faced the possibility of experiencing a stroke, MI, or death in each cycle. As the subsequent event risk and costs were higher in the stroke and post-stroke states, patients could not transition from the non-fatal stroke or post-stroke states to the non-fatal MI or post-MI states. Transition probabilities were based on a previous CEA with similar populations.^10^ Transition probabilities for subsequent events were derived by multiplying baseline probabilities by relative risk factors. Patients in ‘Post-MI’ and ‘Post-stroke’ states had a higher risk of subsequent events than those in the ‘No-event’ state. Mortality rates, based on age-specific data from Dutch population life tables, increased with age. All model inputs are detailed in *Table 1*.

**Table 1:** Model input parameters

Parameters	Base-case value	Range	Distribution	Source
Probabilities (decision tree)				
Standard care				
Minor bleeding	0.166	0.125–0.208	Beta	Azzahhafi *et al.*^9^
Major bleeding	0.042	0.001–0.052	Beta	Azzahhafi *et al.*^9^
MI	0.032	0.024–0.041	Beta	Azzahhafi *et al.*^9^
Stroke	0.017	0.013–0.022	Beta	Azzahhafi *et al.*^9^
All-cause death	0.026	0.019–0.028	Beta	Azzahhafi *et al.*^9^
Genotype-guided treatment				Azzahhafi *et al.*^9^
Minor bleeding	0.106	0.079–0.132	Beta	Azzahhafi *et al.*^9^
Major bleeding	0.0070	0.0055–0.0092	Beta	Azzahhafi *et al.*^9^
MI	0.030	0.022–0.037	Beta	Azzahhafi *et al*.^9^
Stroke	0.015	0.011–0.018	Beta	Azzahhafi *et al.*^9^
All-cause death	0.022	0.017–0.028	Beta	Azzahhafi *et al.*^9^
Probabilities (Markov model)^a^				
Annual risk from ‘No-event’ to ‘MI’	0.019	0.01–0.05	Beta	Nikolic *et al.*^10^
Annual risk from ‘No-event’ to ‘Stroke’	0.003	0.001–0.002	Beta	Nikolic *et al.*^10^
Annual risk from ‘No-event’ to ‘Non-CV death’	Age specific mortality rate		Beta	CBS^37^
Increased risk of a subsequent event after having an event	2.0	1.0–4.0	LOGNORMAL	Lala *et al.*^38^
Increased risk of death in ‘No-event’	2.0	1.5–2.5	LOGNORMAL	Nikolic *et al.*^10^
Increased risk of death in ‘Non-fatal MI’	6.0	4.5–7.5	LOGNORMAL	Nikolic *et al.*^10^
Increased risk of death in ‘post MI’	3.0	2.25–3.75	LOGNORMAL	Nikolic *et al.*^10^
Increased risk of death in ‘Non-fatal stroke’	7.43	5.57–9.29	LOGNORMAL	Nikolic *et al.*^10^
Increased risk of death in ‘post stroke’	3.0	2.25–3.75	LOGNORMAL	Nikolic *et al.*^10^
Costs (in euro's)^b^				
Costs CYP2C19 lab test	75	56.25–93.75	Gamma	Azzahhafi *et al.*^14^
Costs CYP2C19 POCT test	150	112.50–187.50	Gamma	Azzahhafi *et al.*^14^
1 year clopidogrel treatment	51.10	38.33–63.88	Gamma	ZIN^39^
1 year ticagrelor treatment	876.00	657–1095	Gamma	ZIN^40^
1 year prasugrel treatment	478.10	358.61–597.69	Gamma	ZIN^41^
Minor bleeding	321.03	221.68–508.5	Gamma	Jacbos *et al.*^42^
Major bleeding	5601.92	3243.55–9476.25	Gamma	Ten Cate-Hoek *et al.*^43^
MI	5734.33	3320.21–9700.3	Gamma	Soekhlal *et al.*^44^
Post-MI	2620.61	2776.3–3128.85	Gamma	De Jong *et al.*^45^
Stroke	29 166.05	21 554.88–45512.13	Gamma	De Jong *et al.*^45^
Post-stroke	11 932.74	9059.22–17118.81	Gamma	De Jong *et al.*^45^
All-cause death	3558.19	3495.21–3769.77	Gamma	Greving *et al.*^46^
Utilities^c^				
No event	0.838	0.7179–0.927	Beta	FORCE-ACS
Myocardial infarction	0.744	0.66–0.87	Beta	FORCE-ACS
Post-MI	0.744	0.66–0.87	Beta	FORCE-ACS
Stroke	0.620	0.6–0.64	Beta	Nikolic *et al.*^10^
Post-stroke	0.620	0.6–0.64	Beta	Nikolic *et al.*^10^
Death	0	NA	NA	
Minor bleeding (disutility 2 days)	0.073	0.054–0.091	Beta	FORCE-ACS
Major bleeding (disutility 14 days)	0.140	0.07–0.21	Beta	Stevanovic *et al.*^47^

CI, confidence interval; CBS, Central Bureau of Statistics; CV, cardiovascular; NA, not applicable; MI, myocardial infarction; ZIN, Zorginstituut Nederland [National Health Care Institute Netherlands].

a Range indicating min/max as provided by paper. If min/max was unavailable, ranges were calculated with 25% of the base-case value.

b Range is based on 95% CI. If 95% CI was unavailable, ranges were calculated with standard error of 25% of the mean.

c Range is based on 95% CI.

#### Costs

The CEA was performed from the healthcare perspective, and all costs were based on the Dutch healthcare system. Costs were inflated to 2023 using a calculator based on the consumer price index inflation from the Dutch Central Bureau of Statistics (Supplementary material online, *Table S1*).^13^ They consisted of treatment costs of the different antiplatelet drugs, genetic tests, and costs associated with cardiovascular events (minor bleeding, major bleeding, non-fatal MI, non-fatal stroke, post-MI, post-stroke, and death). Based on a previous analysis, de-escalation occurred within 48 h in the majority of patients.^14^ Therefore, the use of ticagrelor, prasugrel, and clopidogrel during the first year was based on the prescribed P2Y12 inhibitor at discharge in both cohorts and the treatment adherence during that year. Unplanned switching between P2Y12 inhibitors occurred frequently, especially from ticagrelor to clopidogrel, and predominantly early, with a median time to switch from ticagrelor to clopidogrel of 65 days and clopidogrel to ticagrelor of 19 days (Supplementary material online, *Table S1*). Therefore, regarding drug costs, we assumed that patients who switched to another P2Y12 inhibitor were treated with the latter P2Y12 inhibitor for the entire year. Both the costs and allocation between the use of a *CYP2C19* POC test (in 88% of patients) and lab test (in 12% of patients) were determined from a prior feasibility analysis of the clinical implementation of a genotype-guided de-escalation strategy.^14^ All costs were discounted using an annual rate of 3% in line with existing Dutch guidelines for health-economic evaluations.^15^

#### Health utilities

Health utilities were quantified in quality-adjusted life years (QALYs) and derived from the FORCE-ACS registry population for minor bleeding, no-event state, MI state, and post-MI state. At 12 months after initial hospital admission, QoL was measured using the 12-item Short Form Survey version 2. EQ-5D results were based on complete SF-12 questionnaire responses, and estimated using the method outlined by Gray *et al.*^16^ Because of the limited number of patients who experienced major bleeding and/or stroke and completed an SF-12 questionnaire at 1 year, we derived the utilities for these events in similar populations from literature.^10,17^

Based on prior literature, bleeding resulted in temporary disutility throughout the first year of the model.^17^ We assumed that adverse events from antiplatelet therapy, like dyspnoea or bruises, did not have long-term prognostic effects on QoL and, therefore, were not accounted for the calculation of utilities for the base-case values.^10^

#### Outcomes

The outcome measures were costs, QALYs, incremental cost-effectiveness ratios (ICERs) expressed in euros per QALY gained, and net monetary benefit (NMB), calculated as (incremental benefit × threshold)—incremental cost. If both incremental costs and QALYs were positive, the ICER was calculated. If both incremental costs and QALYs were negative, NMB was calculated, as the resulting ICER would not be informative.^18^ A positive NMB would indicate that the genotype-guided strategy is cost-effective compared with standard DAPT at the given willingness-to-pay threshold. Since antiplatelet therapy is used for tertiary prevention, we used a reference value of €20 000 per QALY.^19^

#### Sensitivity and scenario analysis

The base-case analysis was based on model inputs shown in *Table 1*. To address model uncertainties, we conducted both univariate deterministic (DSA) and probabilistic sensitivity analyses (PSA). Parameter ranges were based on 95% confidence intervals (CI) or a standard error of 25%. In the univariate DSA, each parameter was varied individually over its 95% CI or fixed range. The PSA employed a Monte Carlo simulation with 10 000 iterations, randomly and simultaneously varying all parameters within their 95% CIs or fixed ranges. The distributions used for each parameter are detailed in *Table 1*.

To evaluate the robustness of the results, scenario analyses were conducted with different time horizons (scenario 1) and by equalizing all prices to mimic the availability of generic versions of ticagrelor and prasugrel (scenario 2). We performed additional analyses to illustrate the impact of decreasing drug prices on cost-efficacy. In the base case model, we used the event rates from the FORCE-ACS registry. Since the confidence intervals showed no difference in ischaemic event rates between the two groups, we conducted a third scenario where ischaemic event rates were identical (scenario 3). Finally, as both minor and major bleeding have been associated with increased morbidity and lower QoL for a prolonged time, a fourth scenario analysis accounted for a prolonged duration of disutility of bleeding (scenario 4).^20–22^

## Results

### Base-case and alternative base case analyses

**Figure 2:**
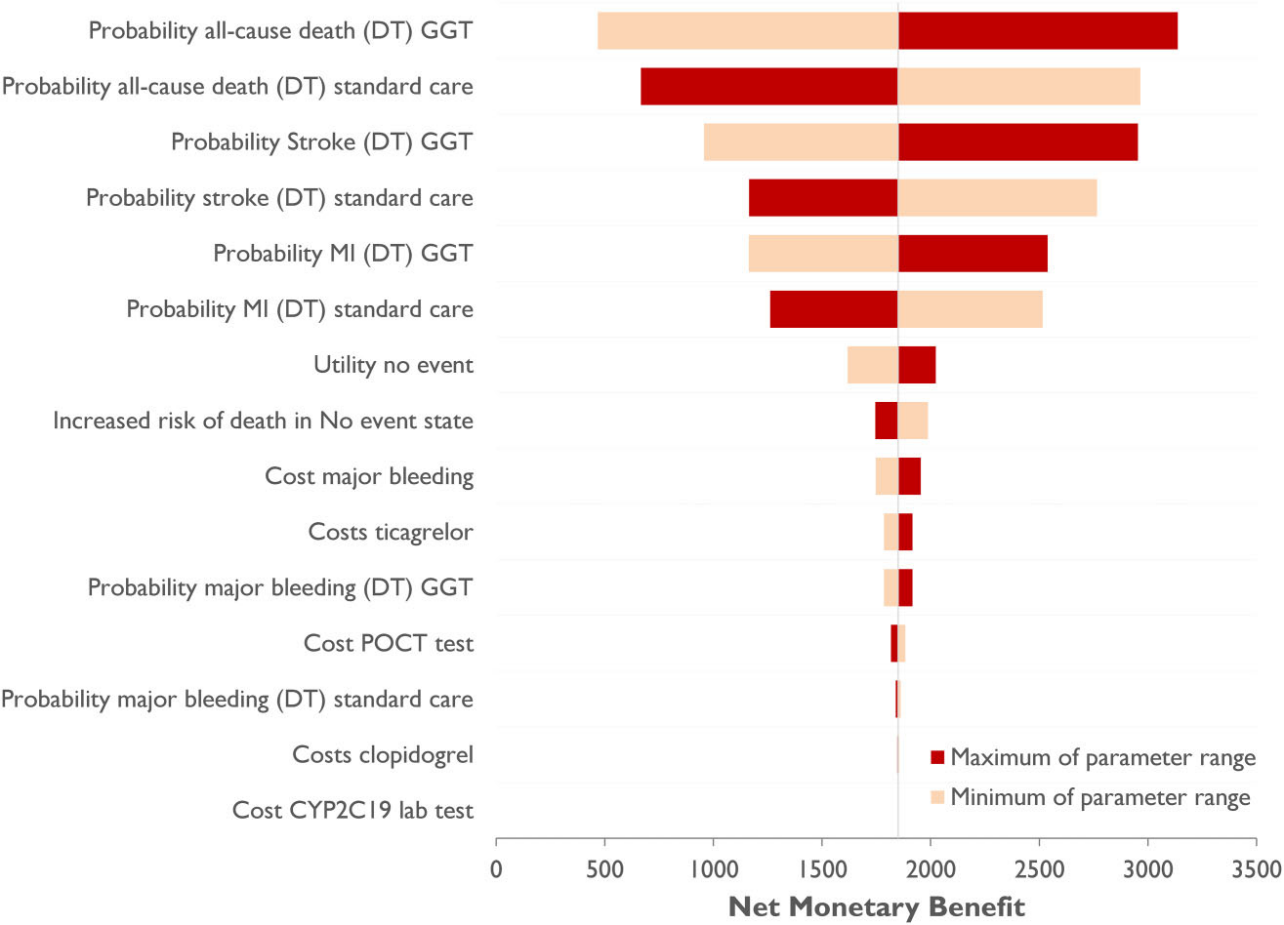
Deterministic sensitivity analysis. Tornado plot showing the net monetary benefit (NMB). In the deterministic sensitivity analysis (DSA), the minimum and maximum value of the parameter range of every individual parameter is alternately put into the model. The results of the DSA depict the influence on the NMB when the minimum or maximum value of the individual parameter is used, while all other parameters stay the same. The base case value of the NBM was 1850.7 DT: decision tree, MI, myocardial infarction.

**Figure 3:**
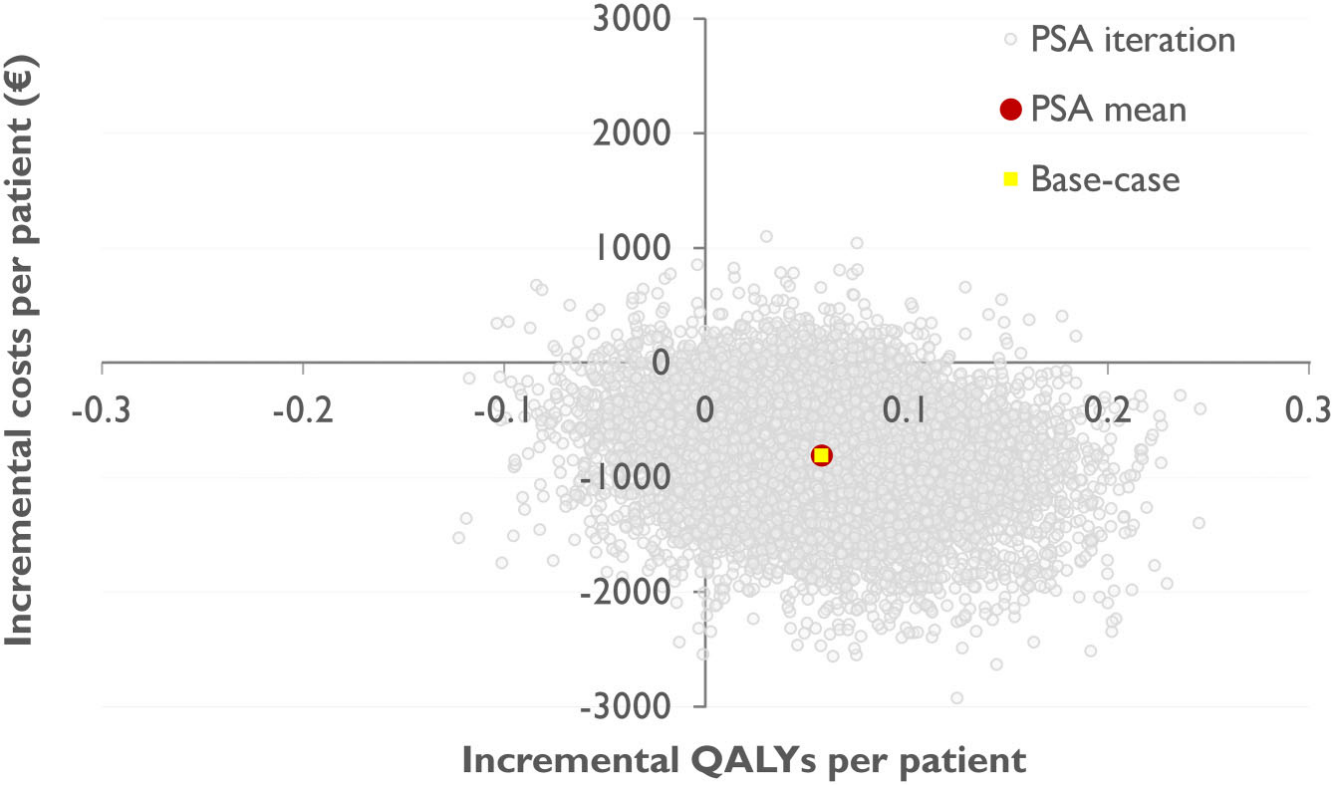
Probabilistic sensitivity analysis. Cost-effectiveness plane showing the results of the probabilistic sensitivity analysis (PSA) demonstrating the varying outcomes of the Monte Carlo analysis, with 10 000 iterations per patient, where all model inputs are randomly adjusted based on their respective uncertainty distributions. Both the average PSA value and the outcome of the base-case scenario are displayed in the figure. QALY, quality-adjusted life year.

Based on a hypothetical cohort of 1000 patients admitted for ACS, a genotype-guided de-escalation strategy resulted in a lifetime increase of 57.30 QALYs, while saving €808 788, compared to standard prescription of DAPT. This equated to an average gain of 0.058 QALYs and €809 saved per patient. The incremental NMB of the genotyped guided strategy was €1962 per patient. The univariate DSA, represented in a tornado plot (*Figure 2*), revealed that the distribution of patients across the different health states by the decision tree (all-cause mortality, MI, and stroke) exerted the most significant impact on the model outcomes. Furthermore, the findings from probabilistic sensitivity analysis (PSA), depicted in a cost-effectiveness plane (*Figure 3*), indicated that treatment with clopidogrel was cost saving in 96% of the 10 000 Monte Carlo simulation iterations, whereas it increased QALYs in 87% of the iterations. In 95% of the iterations, the NMB was higher in the genotype-guided group compared to the standard DAPT group, indicating cost-efficacy.

### Scenario analyses

**Figure 4:**
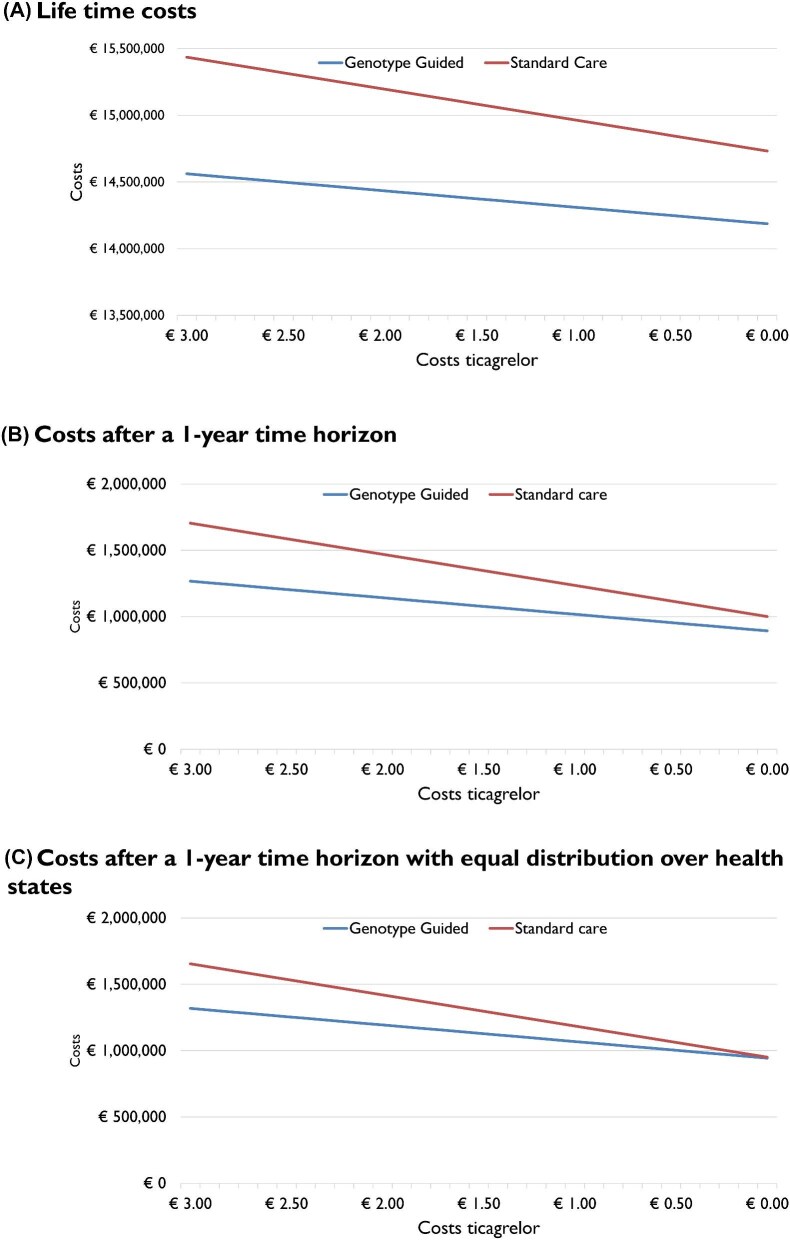
Impact of reducing ticagrelor prices on costs in different scenarios. Results of a scenario analysis demonstrating the impact of reducing ticagrelor prices on total costs in the genotype-guided and standard care cohorts. (*A*) Total costs based on the base-case analysis and a lifetime horizon. (*B*) Total costs based on the base-case analysis and a 1-year horizon. (*C*) Costs based on the scenario with equal distribution over health states and a 1-year horizon.

*Table 2* shows the results of the different scenario analyses. In scenario 1, adjusting the time horizon did not alter the conclusions regarding the cost-effectiveness of the intervention. After 1 year, implementing a de-escalation strategy led to a net cost reduction of −€460 924. This reduction was primarily driven by decreased medication expenses (−€261 723) compared to standard care, despite the costs associated with performing the genetic tests (+€140 965) in the genotype-guided cohort. The intervention remained cost-saving in scenario 2, where prices for all P2Y12-inhibitors were equalized, primarily due to the increased costs associated with higher bleeding rates in the standard care cohort. In addition, when applying this scenario over a 1-year time horizon, the genotype-guided strategy was cost-saving (−€199 202). In *Figure 4*, we illustrated the potential impact of decreasing prices for ticagrelor on cost-savings. Under varying scenarios and time horizons, the genotype-guided strategy was cost-saving compared to standard care with ticagrelor prices ranging from €0 to €3/day. In scenario 3, where ischaemic events rates were identical, costs remained lower in the genotype-guided group. In this scenario, the increase in QALYs (0.20) is attributable to the decrease of bleeding in the intervention group. In the fourth scenario, the period of the disutility of bleeding was extended. This increased QALYs associated with the guided strategy from 57.53 (base case) to 61.76 (182 days) and 66.22 (365 days).

**Table 2:** Lifetime cost-effectiveness results for base-case and scenario analyses

	Costs genotype-guided strategy (€)	Costs standard care (€)	∆Costs (€)	QALYs genotype-guided strategy	QALYs standard care	**∆QALY**	**ICER** ***^a^*** **(€/QALY)**
**Base case**	€14 486 401	€15 295 189	−€808 788	10 555.88	10 498.35	57.73	Dominating
**Scenario analyses**							
**Scenario 1** *Different time horizons*							
*1 year*	€1 245 054	€1 705 979	−€460 924	813.35	809.28	4.07	Dominating
*5 years*	€4 462 851	€5 094 354	−€631 503	4424.88	4402.64	22.23	Dominating
*10 years*	€8 239 353	€8 975 510	−€736 157	7222.22	7185.16	37.06	Dominating
*20 years*	€13 582 781	€14 390 327	−€807 546	10 167.31	10 112.71	54.59	Dominating
**Scenario 2**							
*Identical prices for P2Y_12_ inhibitors*	€14 198 874	€14 745 940	−€547 065	10 555.88	10 498.35	57.53	Dominating
**Scenario 3**							
*Equal distribution over health states for all-cause death, MI and stroke*	€14 713 834	€15 068 187	−€354 353	10 527.22	10 527.02	0.20	Dominating
**Scenario 4** *Prolonged duration of bleeding disutility*							
*182 days**^b^*	€14 486 401	€15 295 189	−€808 788	10 551.71	10 489.95	61.76	Dominating
*365 days**^b^*	€14 486 401	€15 295 189	−€808 788	10 547.44	10 481.22	66.22	Dominating

ICER, incremental cost-effectiveness ratio; NA, not applicable; QALY, quality-adjusted life year.

a When both the incremental costs and QALYs were negative, the ICER could not be calculated.

b Disutility for both BARC 2 (minor bleeding) and BARC 3 bleeding (major bleeding).

## Discussion

This is the first CEA evaluating the economic benefits of a genotype-guided de-escalation strategy using data from its implementation in clinical care. These cost-efficacy data suggest that implementing a genotype-guided de-escalation strategy in clinical practice is associated with an increase in QALYs and a reduction in costs compared to standard DAPT in patients with ACS. Multiple sensitivity and scenario analyses consistently replicated the findings of the base-case analysis, confirming that a genotype-guided strategy dominated standard care, as it was both cost-saving and yielded higher QALYs.

In recent years, numerous strategies have been explored to reduce bleeding risk without compromising ischaemic outcomes in patients undergoing DAPT. Considering the growing body of evidence, the preference may shift toward ticagrelor monotherapy after a brief period of DAPT in the coming years.^23^ However, with the ever-rising costs of healthcare, one should not neglect the impact of longer or more frequent prescription of costly drugs like ticagrelor or prasugrel.^24^ While ticagrelor and prasugrel are the most effective at reducing platelet reactivity in patients with a *CYP2C19* loss-of-function allele compared to standard dose clopidogrel, high-dose clopidogrel also lowers platelet reactivity in these patients and may serve as a low-cost option in clinical settings where ticagrelor or prasugrel are unavailable.^25^ However, this approach is not recommended by clinical guidelines, such as those from CPIC, as clopidogrel doses as high as 300 mg may not fully overcome genotype effects in certain intermediate metabolizers (e.g. those with diabetes) or poor metabolizers.^5^

The POPular Genetics trial was the first large RCT to demonstrate that a genotype-guided de-escalation strategy can reduce bleeding events.^4^ Although there were no significant differences in the combined thrombotic outcome between the two groups, a limitation of this study is that it was not powered to detect non-inferiority for ischaemic events. Nevertheless, similar findings have been reported in other observational studies and a meta-analysis, suggesting that clopidogrel has comparable efficacy to ticagrelor or prasugrel in patients without a loss-of-function allele, but reduced efficacy in intermediate or poor metabolizers and those with a high ABCD-GENE (age, body mass index, chronic kidney disease, diabetes, and CYP2C19 genetic variants) score.^26,27^ These results are reinforced by a network meta-analysis indicating that guided selection of P2Y12-inhibitor therapy in ACS patients offers a better balance of safety and efficacy than routine potent P2Y12-inhibitor therapy.^28^

Our results are in line with the CEA of the POPular Genetics, which demonstrated the cost-efficacy of a *CYP2C19* genotype-guided strategy based on data from a randomized trial.^29^ Despite higher overall costs in both groups, which can be attributed to inflation and increased rates of ischaemic events, the incremental cost-savings from both base-case analyses were comparable (FORCE-ACS: −€698,286 vs. POPular Genetics: −€725 551). The increase in incremental QALYs was more pronounced in our analysis (FORCE-ACS: 57.73 vs. POPular Genetics: 8.98), which may be due to the larger disparity in event rates used in the base case. In the PSA cost-effectiveness plane, the POPular Genetics study shows more iterations skewed toward the southeast quadrant, indicating greater cost-effectiveness. Unlike our study, their CEA lacked specified probability ranges for decision tree variables, which may explain the differences. Since our study was not powered to detect event differences, we took a more conservative approach by incorporating uncertainty around the event rates in our model. The tornado plot in *Figure 2* shows that changes in the probabilities used in the decision tree during the initial cycle exert the largest impact on the lifetime outcome of the model. Despite this conservative approach, the genotype-guided strategy saved costs and was associated with more QALYs gained in 84% of the iterations.

Several studies have explored the cost-effectiveness of *CYP2C19* genotype-guided strategies. However, none have used data from a study where a de-escalation strategy was implemented.^30–32^ In a secondary analysis, Limdi *et al.* assessed the cost-efficacy of a genotype-guided de-escalation applied 30 days post-PCI. They found it was not cost-effective (ICER of $188 680/QALY), but resulted in a higher NMB than universal use of ticagrelor. An important constraint is that this analysis relied on data from an escalation strategy, rather than a de-escalation strategy, making it challenging to assess cost-effectiveness for a de-escalation approach. A CEA based on the Veterans Health Administration showed that a combined approach of genotype-guided escalation and de-escalation strategies can improve cardiovascular outcomes and reduce costs within 12 months.^33^ However, the analysis relied on RCT data for treatment effects rather than real-world data, which may limit the generalizability of the findings. Notably, the study emphasized that health systems should prioritize high adherence to the de-escalation strategy, as it was the primary driver of cost-effectiveness.

Our analysis benefits from using prospectively registered real-world data, allowing us to account for adherence to the de-escalation protocol and P2Y12-inhibitor therapy in the first year after ACS. Instead of assuming universal de-escalation to clopidogrel, we considered that only 89% did so, aligning with prior data.^14^ We also adjusted the standard care cohort to reflect that only 64% received ticagrelor. These considerations lead to more conservative results, but ones that are closer to clinical practice.

With the anticipated expiration of the patent for ticagrelor, prices are expected to gradually decrease in the coming years. We demonstrated that even with decreasing ticagrelor prices, a genotype-guided de-escalation strategy remains cost-effective, as the beneficial effect on bleeding can offset these lower prices.

Our findings, alongside results from RCTs and consensus recommendations, should prompt guideline committees to provide stronger recommendations on the use of genetic testing in clinical practice, as current guidelines either omit this strategy or offer only weak guidance.^34–36^

### Limitations

Our analysis is subject to several limitations. First, as the FORCE-ACS registry could only provide data regarding treatment and outcomes during the first year, we had to make assumptions based on other data to estimate long-term cost-effectiveness. However, the majority of these assumptions are based on data from comparable populations and similar clinical settings. Second, the probabilities in the decision tree were derived from observational data comparing two cohorts enrolled during different time periods. Since this analysis was not powered to detect differences in ischaemic and bleeding rates, further research with a larger sample size is required for more conclusive results. Third, as less than 1% of patients were treated with prasugrel, our findings are mainly relevant to treatment with clopidogrel and ticagrelor. Fourth, while converting SF-12 data to EQ-5D responses is pragmatic given the data constraints, it introduces some uncertainty in the precision of QALY calculations. Fifth, our analysis used a healthcare perspective, though a societal perspective is preferable. This would include non-healthcare costs and productivity loss, which we could not account for, as we did not register this data. Finally, while our results advocate for the cost-effectiveness of genotype-guided de-escalation, the routine implementation of CYP2C19 genotyping may vary based on local infrastructure and associated costs, which can differ across healthcare settings in different countries.

## Conclusion

A genotype-guided de-escalation strategy in patients with ACS dominated standard DAPT consisting of aspirin plus ticagrelor/prasugrel by being cost-saving and yielding higher QALYs. These findings underscore the cost-effectiveness of implementing a genotype-guided de-escalation strategy into clinical practice.

## Supplementary Material

pvae087_Supplemental_File

## Notes

**Conflict of interest**: J.P.S.H. has received institutional research grants from Abbott Vascular, AstraZeneca, B. Braun, Getinge, Ferrer, Infraredx, and ZonMw. W.J.K. has received an institutional research grant from AstraZeneca. J.M.B. has received institutional research grants from AstraZeneca, Daiichi Sankyo, and ZonMw and personal fees from AstraZeneca, Bayer, Boehringer Ingelheim, CeleCor Therapeutics, Daiichi Sankyo. All other authors have no relationships with industry to disclose.

## Data availability

The data underlying this article will be shared on reasonable request to the corresponding author.
