# Supplementary material for: Cost-effectiveness of implementing a genotype-guided de-escalation strategy in patients with acute coronary syndrome
Source: Eur Heart J Cardiovasc Pharmacother. 2024 Nov 13;11(3):230–40. doi: 10.1093/ehjcvp/pvae087 (PMC12046577; doi:10.1093/ehjcvp/pvae087)
Supplement: pvae087_Supplemental_File [file pvae087_supplemental_file.docx]

**Supplementary data**

**Cost-effectiveness of Implementing a Genotype-Guided De-Escalation Strategy in Patients with Acute Coronary Syndrome**

Table of contents

[Table S1. Baseline and treatment characteristics 2](#_Toc169769008)

**Tables**

# Table S1. Baseline and treatment characteristics

| **Patients characteristics** | **Genotyped cohort**  (N = 406) | **Standard care cohort**  (N = 1203) |
| --- | --- | --- |
| Age in years, median (IQR) | 64.00 [55.25, 73.00] | 64.00 [56.00, 73.00] |
| Female sex, n (%) | 115 (28.3) | 340 (28.3) |
| BMI, mean (SD) | 27.83 (5.00) | 27.61 (4.30) |
| Current smoking, n (%) | 115 (28.3) | 340 (28.3) |
| Hypertension, n (%) | 27.83 (5.00) | 27.61 (4.30) |
| Hypercholesterolemia, n (%) | 144 (35.5) | 382 (31.8) |
| Previous MI | 57 (14.0) | 172 (14.3) |
| Previous PCI | 57 (14.0) | 177 (14.7) |
| STEMI at admission | 234 (57.6) | 676 (56.2) |
| NSTEMI at admission | 147 (36.2) | 461 (38.3) |
| **Procedural characteristics** | | |
| CAG, n (%) | 392 (96.6%) | 1171 (97.3) |
| PCI, n (%) | 333 (82.0%) | 978 (81.3) |
| CABG, n (%) | 29 (7.3%) | 121 (10.1) |
| Acetylsalicylic acid, n (%) | 406 (100%) | 1203 (100%) |
| Clopidogrel, n (%) | 241 (59.4%) | 219 (18.2) |
| Ticagrelor, n (%) | 164 (40.4%) | 978 (81.3) |
| Prasugrel, n (%) | 1 (0.2) | 6 (0.5) |
| **Treatment alterations** | | |
| Clopidogrel to ticagrelor | 4 (1.0%) | 10 (0.8%) |
| Ticagrelor to clopidogrel | 25 (6.2%) | 167 (13.9%) |
| Ticagrelor to prasugrel | 4 (1.0%) | 48 (4.0%) |

CABG = coronary artery bypass grafting; CAG = coronary angiography; IQR = interquartile range; MI = myocardial infarction; NSTEMI = non-ST-segment elevation myocardial infarction; PCI = percutaneous coronary intervention; SD = standard deviation; STEMI = ST-segment elevation myocardial infarction.

# 
